# Supplementary material for: Dynamic transcriptomic profiles of zebrafish gills in response to zinc supplementation
Source: BMC Genomics. 2010 Oct 11;11:553. doi: 10.1186/1471-2164-11-553 (PMC3091702; doi:10.1186/1471-2164-11-553)
Supplement: Additional file 2 — Interactive Direct Interaction Network representing the molecular interactions between zinc, copper, iron, calcium and proteins encoded by transcripts changed by zinc supplementation. Mini web-site containing index.html and hyperlinked pages in subdirectory describing a Direct Interaction Network automatically generated based on curated interactions contained within the proprietary PathwayArchitect database. Ovals represent proteins and the circles symbolize metal ions. Objects are coloured by their abundance in zebrafish at the time-point they were significantly different from the control is a scale from -4 fold (dark green) to +4 fold (dark red). Where significant differences were found at more than one time-point, the colour overlay shows expression at the first instance. Dark blue squares denote 'binding', and light blue squares 'expression'; green squares stand for 'regulation', green diamonds for 'metabolism', and green circles for 'promoter binding'. Arrow heads indicate directionality of the interaction where annotated. All nodes and edges can be further interrogated by selecting the relative area of the image. [file 1471-2164-11-553-S2.zip › PathwayArchitect Zn xs DIN/402689.html]

# REGULATION:

|  |  |
| --- | --- |
| Type | REGULATION |
| Effect | Positive |


---

|  |  |
| --- | --- |
| Score | 0 |


---

|  |  |
| --- | --- |
| Reference Count | 14 |


---

|  |  |
| --- | --- |
| Mechanism | Unknown |


---

|  |  |
| --- | --- |
| Reference:0 || Sentence | "Thus, regulation of Cu-Zn SOD mRNA levels contribute to life stage differences in copper distribution." |
| PMID | 8427881 |
| Year | 1993 |
| Species | Rat |
| Journal | Biochim Biophys Acta |
| RefScore | 1 |
| Source | PArchNLP |
  |
|  |  |


---

|  |  |
| --- | --- |
 Reference:1 || Sentence | "It is suggested that zinc interferes with copper absorption by inducing MT, which sequesters copper in the mucosal cells, making it unavailable for serosal transfer." |
| PMID | 6822916 |
| Year | 1983 |
| Species | Rat |
| Journal | J Nutr |
| RefScore | 1 |
| Source | PArchNLP |
  ||  |  |


---

|  |  |
| --- | --- |
 Reference:2 || Sentence | "Trientine gives a strong, fast, negative copper balance, and zinc induces hepatic metallothionein, which sequesters hepatic copper." |
| PMID | 8521757 |
| Year | 1995 |
| Species | Human |
| Journal | Drugs |
| RefScore | 1 |
| Source | PArchNLP |
  ||  |  |


---

|  |  |
| --- | --- |
 Reference:3 || Sentence | "This protein, termed copper chelatin or thionein, is related to the metallothionein family of proteins that are induced in response to cadmium and zinc in vertebrate cells." |
| PMID | 6374656 |
| Year | 1984 |
| Journal | Proc Natl Acad Sci U S A |
| RefScore | 1 |
| Source | PArchNLP |
  ||  |  |


---

|  |  |
| --- | --- |
 Reference:4 || Sentence | "Copper absorption was enhanced by the dietary levels of riboflavine, cellulose, milk proteins, oxalates and zinc, while phosphorus, niacin, calcium and pulse protein inhibited the absorption of copper." |
| PMID | 8185283 |
| Year | 1994 |
| Species | Human |
| Journal | Ann Nutr Metab |
| RefScore | 1 |
| Source | PArchNLP |
  ||  |  |


---

|  |  |
| --- | --- |
 Reference:5 || Sentence | "After zinc supplementation, serum copper concentration decreased but there was no change in the ratio of low-density-lipoprotein cholesterol to high-density-lipoprotein cholesterol." |
| PMID | 8460613 |
| Year | 1993 |
| Species | Human |
| Journal | Am J Clin Nutr |
| RefScore | 0 |
| Source | PArchNLP |
  ||  |  |


---

|  |  |
| --- | --- |
 Reference:6 || Sentence | "CONCLUSIONS: Head neck cancer patients show the same characteristic trace element status as other solid tumors: decreased selenium, zinc and iron, increased copper." |
| PMID | 12915990 |
| Year | 2003 |
| Species | Human |
| Journal | Laryngorhinootologie |
| RefScore | 1 |
| Source | PArchNLP |
  ||  |  |


---

|  |  |
| --- | --- |
 Reference:7 || Sentence | "IL-1 reduced zinc and iron plasma levels and elevated copper plasma levels." |
| PMID | 8018447 |
| Year | 1993 |
| Species | Mouse |
| Journal | Biotherapy |
| RefScore | 1 |
| Source | PArchNLP |
  ||  |  |


---

|  |  |
| --- | --- |
 Reference:8 || Sentence | "Zinc acts by inducing intestinal cell metallothionein, which binds copper with high affinity, blocking its absorption, and causing its excretion in the stool." |
| PMID | 2258535 |
| Year | 1990 |
| Species | Human |
| Journal | J Am Coll Nutr |
| RefScore | 0 |
| Source | PArchNLP |
  ||  |  |


---

|  |  |
| --- | --- |
 Reference:9 || Sentence | "Seven subjects (6.5%) fell below the criteria for low serum zinc levels (< 11.5 mumol.l-1) while two subjects (1.8%) were below the criteria for low serum calcium levels (< 2.20 mmol.l-1). 2) Therapeutic oral iron supplementation was successful in raising mean serum ferritin values from 15.9 micrograms.l-1 to 36.5 micrograms.l-1 but was not associated with decrements in serum copper or calcium levels. 3) The treatment did not significantly effect serum zinc and magnesium levels during the supplementation period, but a downward trend continued through the discontinuation phase so that at 18 and 24 wk serum zinc and magnesium levels were significantly lower than baseline. 4) Oral contraceptive use was associated with elevated serum copper and ferritin values and lowered serum magnesium levels." |
| PMID | 8492683 |
| Year | 1993 |
| Species | Human |
| Journal | Med Sci Sports Exerc |
| RefScore | 4 |
| Source | PArchNLP |
  ||  |  |


---

|  |  |
| --- | --- |
 Reference:10 || Sentence | "The present study was conducted to assess in rats the effects of oral aluminum (Al) exposure on calcium (Ca), magnesium (Mg), manganese (Mn), copper (Cu), zinc (Zn), and iron (Fe) accumulation and urinary excretion." |
| PMID | 11318238 |
| Year | 2001 |
| Species | Rat |
| Journal | Biol Trace Elem Res |
| RefScore | 1 |
| Source | PArchNLP |
  ||  |  |


---

|  |  |
| --- | --- |
 Reference:11 || Sentence | "The beneficial effects of S-adenosyl-L-methionine (SAM) in potentiating the mobilization of cadmium by cadmium trisodium diethylenetriamine penta acetic acid (DTPA) from the major target organs and restoration of depleted tissue glutathione (GSH), zinc and copper concentration, were determined in cadmium-exposed mice." |
| PMID | 11258544 |
| Year | 2001 |
| Species | Mouse |
| Journal | Cytobios |
| RefScore | 1 |
| Source | PArchNLP |
  ||  |  |


---

|  |  |
| --- | --- |
 Reference:12 || Sentence | "Accumulation of zinc in liver and their copper and ceruloplasmin production also elicits increase of copper and decrease of zinc in gingiva." |
| PMID | 8502198 |
| Year | 1993 |
| Species | Human |
| Journal | Med Hypotheses |
| RefScore | 1 |
| Source | PArchNLP |
  ||  |  |


---

|  |  |
| --- | --- |
 Reference:13 || Sentence | "These results indicate that zinc binding to histatin 5 involves His-15 present within the -H-E-X-X-H- zinc binding motif, and copper binding occurs within the N-terminal D-S-H-, ATCUN motif." |
| PMID | 11226423 |
| Year | 2001 |
| Species | Human |
| Journal | FEBS Lett |
| RefScore | 3 |
| Source | PArchNLP |
  |


---

|  |  |
| --- | --- |
